# The Optimal Licensing Contract in a Differentiated Stackelberg Model

**DOI:** 10.1155/2014/437919

**Published:** 2014-02-10

**Authors:** Xianpei Hong, Lijun Yang, Huaige Zhang, Dan Zhao

**Affiliations:** ^1^School of Economics and Management, Hubei University of Automotive Technology, Shiyan 442002, China; ^2^School of Management, Huazhong University of Science & Technology, Wuhan 430074, China; ^3^Antai College of Economics & Management, Shanghai Jiao Tong University, Shanghai 200052, China

## Abstract

This paper extends the work of Wang (2002) by considering a differentiated Stackelberg model, when the leader firm is an inside innovator and licenses its new technology by three options, that is, fixed-fee licensing, royalty licensing, and two-part tariff licensing. The main contributions and conclusions of this paper are threefold. First of all, this paper derives a very different result from Wang (2002). We show that, with a nondrastic innovation, royalty licensing is always better than fixed-fee licensing for the innovator; with a drastic innovation, royalty licensing is superior to fixed-fee licensing for small values of substitution coefficient *d*; however when *d* becomes closer to 1, neither fee nor royalty licensing will occur. Secondly, this paper shows that the innovator is always better off in case of two-part tariff licensing than fixed-fee licensing no matter what the innovation size is. Thirdly, the innovator always prefers to license its nondrastic innovation by means of a two-part tariff instead of licensing by means of a royalty; however, with a drastic innovation, the optimal licensing strategy can be either a two-part tariff or a royalty, depending upon the differentiation of the goods.

## 1. Introduction

As an important way to realize the technology commercialization strategy, technology licensing exerts important influence on improving products' market competitiveness, increasing innovation incentives, and enhancing innovation capability. In some technology-intensive industries, technology innovation and technology licensing are gaining increasing attention among firms and have become part of firm's strategic management. In computer industry, for example, the profit of IBM by technology licensing reached $1.3 billion (10% of its pretax profits) in 2000. Another example is Texas Instruments in semiconductor industry which gains a number of $40 million annually from patent licensing [1].

In order to gain a competitive edge, many firms with weak R&D ability and insufficient fund to engage in R&D activity prefer achieving the license for a new technology from an innovating firm, rather than expending the time and money on developing its own technology [2]. In exchange, the licensee usually needs to make payments under one of the following forms: (1) a fixed fee that is invariant free from the influence of the quantity produced with the new technology; (2) a royalty that depends on the licensee's production quantity using the new technology; and (3) a two-part tariff, that is, a hybrid license consisting of a fixed fee and a per-unit royalty. Empirical studies prove that fixed-fee, royalty, and two-part tariff licensing are popular licensing methods in practice. In a survey of technology license, Rostoker [3] finds that fixed fees alone account for 13 percent of the licensing contracts, royalties alone account for 39 percent, and two-part tariff for 46 percent. In another survey, Macho-Staler et al. [4] shows that 25.3 percent of licensing contracts used fixed fees alone, 62.25 percent royalties alone, and 12.5 percent down payments plus a running royalty.

The literature on technology licensing has developed mainly along three lines. One strand investigates the optimal licensing contract by assuming that the patentee is an outsider. This strand of the literature includes Kamien and Truman [5], Kabiraj [6], Sen [7], Crama et al. [8], Rey and Salant [9] and Chang et al. [10]. For example, Kamien and Truman [5] discuss the outsider innovator's fixed-fee licensing and royalty licensing towards several downstream homogeneous Cournot competitive firms and draw a conclusion that fixed-fee licensing is better than royalty licensing. Chang et al. [10] consider the vertically related market structure where the outside patentee transfers a cost-reducing technology to one or several downstream firms by means of either a fixed fee or a royalty. Sen [7] shows that it is the incomplete information of the incumbent monopoly firm's cost that leads to a diversification in optimal licensing mechanisms, which can also explain the coexistence of a variety of licensing mechanisms in practice.

The second strand of the literature examines the optimal licensing contract by assuming that the patentee is an insider. This strand of the literature includes Wang [11, 12], Matsumura and Matsushima [13], Kishimoto and Muto [14], Wang et al. [15], Rockett [16], Kulatilaka and Lin, [17] and Arya and Mittendorf [18]. For example, Kishimoto and Muto [14] investigate a Cournot duopoly market in which the licensor negotiates with the licensee about payments for licensing a cost-reducing innovation. They find that, in the case of a nondrastic innovation, royalty licensing has an advantage over fixed-fee licensing. Wang et al. [15] extend Poddar and Sinha's [19] work to an oligopolistic model consisting of three cost differential firms in a Cournot framework. They show that fixed-fee licensing is better than royalty licensing and two-part tariff licensing if the licensee has a relative production cost advantage, and the optimal licensing contract is royalty or two-part tariff licensing if the licensor has a relative production cost advantage. Arya and Mittendorf [18] investigate the licensing problem from the perspective of supply chain coordination. They show that downstream innovator's royalty licensing to potential entrants can reduce the loss of supply chain efficiency resulting from “double marginalization,” while the fixed-fee licensing cannot function in the same way.

The third strand of the literature examines the cases where the licensor can be either an outsider or an insider. This strand of the literature includes Kamien and Tauman [20], Liao and Sen [21], and Sen and Tauman [22]. For example, Kamien and Tauman [20] show that an outside patentee's profitable licensing strategy is to license its cost-reducing inventions to monopolistic industries, while an inside patentee prefers licensing its inventions to competitive industries.

All of the above papers are only concerned with one or two forms of licensing. Our work differs by considering three forms of licensing simultaneously. Issues related to the study of the present paper have been investigated also by Wang [12], but the author considers a duopoly differentiated Cournot model and only focuses on comparing fixed-fee licensing and royalty licensing from the viewpoint of the patent-holding firm. The purpose of this paper is to extend the duopoly differentiated Cournot model considered by Wang [12] to a duopoly differentiated Stackelberg model, and we also study and compare three popular licensing methods. We find that the main result in this paper is very different from Wang [12]. After involving sequential decisions in the model of Wang [12], we get a very different result that, with a nondrastic innovation, royalty licensing is always better than fixed-fee licensing for the inside innovator.

The outline of this paper is as follows. In Section 2, we describe the model and derive nonlicensing status quo for the two-firm case. Sections 3, 4, and 5, respectively, investigate fixed-fee, royalty, and two-part tariff licensing contract. In Section 6, we compare the abovementioned three means of licensing. We conclude this paper in Section 7.

## 2. The Model and the Case of No Licensing

Consider an industry consisting of two firms, a leader (firm 1) and a follower (firm 2), that produce differentiated products and engage in Stackelberg quantity competition in the market. Suppose firm 1 has achieved a licensable innovation.

For the development of the mathematical models, the following assumptions are adopted.

**Assumption 1**

The inverse demand functions are linear and given by

(1) $$\begin{array}{c} p_{1}=a-\left(q_{1}+dq_{2}\right),\;\;p_{2}=a-\left(q_{2}+dq_{1}\right), \end{array}$$

where *p*_*i*_ and *q*_*i*_  (*i* = 1, 2) represent firm *i*'s price and output, respectively. *d* ∈ (0,1) denotes the degree of the differentiation of the products. Firms produce at constant marginal cost *c*_*i*_, where 0 < *c*_*i*_ < *a*, *i* = 1, 2.

**Assumption 2**

Two firms have the same initial unit production cost, that is, *c*_1_ = *c*_2_ = *c*.

**Assumption 3**

We assume that licensing can decrease the licensee's marginal cost to the same level as the licensor.

In the following sections, we will consider the innovation process by firm 1 that lowers its unit production cost by the amount of *ε*. If firm 1 licenses its technology to firm 2, it will also lower firm 2's unit production cost by the amount of *ε*.

**Assumption 4**

We suppose that, in the licensing process, when firm 2 is indifferent about accepting firm 1's licensing offer and rejecting it, it chooses to accept the offer.

In addition, in the following sections, we will put forward some other assumptions where the need arises to ensure a reasonable model.

With the above notations and assumptions, two firms' profits are given by

(2) $$\begin{array}{c} \pi_{1}=\left(a-q_{1}-dq_{2}-c_{1}\right)q_{1},\\ \pi_{2}=\left(a-q_{2}-dq_{1}-c_{2}\right)q_{2}. \end{array}$$

Solving the duopoly problem with backward induction, we can easily derive the firms' Stackelberg equilibrium quantities and their profits:

(3) q1=2(a−c1)−(a−c2)d2(2−d2),q2=(4−d2)(a−c2)−2(a−c1)d4(2−d2),

(4) π1=[2(a−c1)−(a−c2)d]28(2−d2),π2=[(4−d2)(a−c2)−2(a−c1)d]216(2−d2)2.

Equations (3) and (4) can serve as a reference for deriving results for the alternative licensing models studied in the following sections.

Now, let us consider the case where the patent licensing is absent. Under this condition, the unit production cost for firm 1 is *c*_1_ = *c* − *ε* and for firm 2 is *c*_2_ = *c*. Depending on the magnitude of the innovation *ε*, the cost-reducing innovation can be divided into two separate cases: nondrastic and drastic innovation. According to the definition of Wang [11, 12], when the innovator's innovation size is large enough, then a firm will be driven out of the market if licensing does not occur, that is, the drastic innovation case. On the contrary, nondrastic innovation means each firm has positive level of output even if the innovator does not license its innovation. From (3), under the nondrastic innovation, that is, *ε* < (*a* − *c*)(4 − *d* ^2^ − 2*d*)/2*d*, the market structure is duopoly. Under the drastic innovation condition, that is, *ε* ≥ (*a* − *c*)(4 − *d* ^2^ − 2*d*)/2*d*, the market structure is monopoly (firm 2 is driven out of the market because of no licensing).

### 2.1. Nondrastic Innovation Case

Under a nondrastic innovation condition, that is, *ε* < (*a* − *c*)(4 − *d* ^2^ − 2*d*)/2*d*, both firms will be active in the market even if no licensing occurs between them. Substituting *c*_1_ = *c* − *ε* and *c*_2_ = *c* into (3) and (4) yields (the superscript NL stands for the case of “no licensing”)

(5) q1NL=(2−d)(a−c)+2ε2(2−d2),q2NL=(4−d2−2d)(a−c)−2εd4(2−d2),

(6) π1NL=[(2−d)(a−c)+2ε]28(2−d2),π2NL=[(4−d2−2d)(a−c)−2εd]216(2−d2)2.

### 2.2. Drastic Innovation Case

Under a drastic innovation condition, that is, *ε* ≥ (*a* − *c*)(4 − *d* ^2^ − 2*d*)/2*d*, firm 2 will be driven out of the market if firm 1 does not license its innovation. When technology licensing is absent, firm 1 becomes a monopolist. Solving the monopoly problem yields the following quantities and profits:

(7) $$\begin{array}{c} q_{1}^{\text{NL}}=\frac{a-c+\varepsilon }{2},\;\;q_{2}^{\text{NL}}=0, \end{array}$$

(8) $$\begin{array}{c} \pi_{1}^{\text{NL}}=\frac{{\left(a-c+\varepsilon \right)}^{2}}{4},\;\;\pi_{2}^{\text{NL}}=0. \end{array}$$

In the following sections, we consider, respectively, three licensing methods: fixed-fee licensing, royalty licensing, and two-part tariff licensing. We will then compare these three licensing methods from the viewpoint of the licensor.

In case there will be a technology licensing between the two firms, the game played by them is a non-cooperative four-stage game. In the first stage, the innovator (firm 1) decides whether to license its innovation or not; if it decides to license it, then firm 1 makes a take-it-or-leave-it offer and charges a payment from firm 2. In the second stage, firm 2 decides whether to accept or reject the offer provided by firm 1. In the third stage, firm 1 chooses the output to maximize its profits; and in the last stage, firm 2, being aware of firm 1's output, decides to produce the output.

## 3. Fixed-Fee Licensing

In this section, we consider licensing by means of a fixed-fee only. Under this licensing method, firm 1 licenses its new technology to firm 2 at a fixed fee *F* and then firm 2 can produce as many units as it wishes using the new technology. In the case that licensing occurs, both firms have the same unit production cost, that is, *c*_1_ = *c*_2_ = *c* − *ε*. Imposing *c*_1_ = *c*_2_ = *c* − *ε* into (3) and (4) yields the firms' equilibrium quantities and profits (the superscript *F* stands for the case of “fixed-fee licensing”):

(9) q1F=(2−d)(a−c+ε)2(2−d2),q2F=(4−d2−2d)(a−c+ε)4(2−d2),

(10) π1F=[(2−d)(a−c+ε)]28(2−d2),π2F=[(4−d2−2d)(a−c+ε)]216(2−d2)2.

In the following subsections, we will consider the cases of nondrastic innovation and drastic innovation, respectively.

### 3.1. Nondrastic Innovation Case

With a nondrastic innovation (i.e., *ε* < (*a* − *c*)(4 − *d* ^2^ − 2*d*)/2*d*), the maximum fee firm 1 can charge is such that firm 2's profits equal its no licensing payoff *π*_2_^NL^; that is,

(11) F=π2F−π2NL=[(4−d2−2d)(a−c+ε)]216(2−d2)2 −[(4−d2−2d)(a−c)−2dε]216(2−d2)2.

Under fixed-fee licensing, firm 1's total profit (market profits plus fixed fee) is

(12) π1F+F=[(2−d)(a−c+ε)]28(2−d2) +[(4−d2−2d)(a−c+ε)]216(2−d2)2 −[(4−d2−2d)(a−c)−2dε]216(2−d2)2.

Comparing (12) and (6), we obtain that *π*_1_^*F*^ + *F* > *π*_1_^NL^ if and only if the following condition is satisfied:

(13) (d4−12d3+4d2+32d−16)ε  <2(16−16d−4d2+6d3−d4)(a−c).

For 0 < *d* < 1, it is easy to verify that 16 − 16*d* − 4*d* ^2^ + 6*d* ^3^ − *d* ^4^ > 0. If *d* ≤ 0.5160 then *d* ^4^ − 12*d* ^3^ + 4*d* ^2^ + 32*d* − 16 ≤ 0; hence (13) is automatically satisfied. So we can easily get *π*_1_^*F*^ + *F* ≥ *π*_1_^NL^. For 0.5160 < *d* ≤ 0.7801, it is easy to verify that ((2(16 − 16*d* − 4*d* ^2^ + 6*d* ^3^ − *d* ^4^))/(*d* ^4^ − 12*d* ^3^ + 4*d* ^2^ + 32*d* − 16))(*a* − *c*)<((4 − *d* ^2^ − 2*d*)/2*d*)(*a* − *c*); hence (13) is implied by the fact that the innovation is nondrastic. For 0.7801 < *d* < 1, firm 1 will not license its innovation to firm 2 because the licensing revenue from fixes-fee licensing cannot make up to the loss for firm 1.

### 3.2. Drastic Innovation Case

With a drastic innovation (i.e., *ε* ≥ (*a* − *c*)(4 − *d* ^2^ − 2*d*)/2*d*), by (8) and (10), the maximum fee firm 1 can charge is given by

(14) $$\begin{array}{c} F=\pi_{2}^{F}-\pi_{2}^{\text{NL}}=\frac{{\left[\left(4-d^{2}-2d\right)\left(a-c+\varepsilon \right)\right]}^{2}}{16{\left(2-d^{2}\right)}^{2}}. \end{array}$$

Under fixed-fee licensing, firm 1's total profit (market profits plus fixed fee) is

(15) π1F+F =[2(2−d2)(2−d)2−(4−d2−2d)2](a−c+ε)216(2−d2)2.

Comparing (10) and (20), we obtain that

(16) π1F+F−π1NL =[(2−d2)d(−8+6d)−(4−d2−2d)2](a−c+ε)216(2−d2)2<0.

Hence, for any substitution coefficient *d*, under a fixed fee, firm 1 will not license a drastic innovation to firm 2, and it will become a monopolist.

In accordance with the above analysis, we have the following.

**Lemma 5**

Under a fixed fee, firm 1 will license a nondrastic innovation to firm 2 if and only if condition (13) is satisfied. In particular, firm 1 will license its nondrastic innovation to firm 2 if 0 < *d* ≤ 0.7801 and will not license it if *d* > 0.7801. With a drastic innovation, licensing will not occur.

Lemma 5 implies that, under fixed-fee licensing, the innovator (firm 1) is likely to license its nondrastic innovation as the two products are more distant substitutes. In particular, if 0 < *d* ≤ 0.7801 then it is more profitable for firm 1 to license its nondrastic innovation to firm 2. With a drastic innovation, firm 1 will keep the innovation for its own use and become a monopoly. This result is very different from Wang [12]. Wang [12] shows that firm 1 will license a drastic innovation to firm 2 if two goods are more distant substitutes (0 < *d* ≤ 0.8284) and will not license it if *d* > 0.8284.

## 4. Royalty Licensing

We consider in this section licensing by means of a royalty only. Under this method the leader (firm 1) licenses its new technology to the follower (firm 2) at a fixed royalty rate and the amount of royalty firm 1 gains will depend on the quantity firm 2 will produce using the new technology. In the case that licensing occurs, firm 1's unit production cost is *c*_1_ = *c* − *ε* and firm 2's unit production cost is *c*_2_ = *c* − *ε* + *r*. Substituting *c*_1_ = *c* − *ε* and *c*_2_ = *c* − *ε* + *r* into (3) and (4) yields the firms' equilibrium quantities and profits (the superscript *R* denotes the case of “royalty licensing”):

(17) q1R=(2−d)(a−c+ε)+rd2(2−d2),q2R=(4−d2−2d)(a−c+ε)−(4−d2)r4(2−d2),π1R=[(2−d)(a−c+ε)+rd]28(2−d2),π2R=[(4−d2−2d)(a−c+ε)−(4−d2)r]216(2−d2)2.

Firm 1's total income is

(18) π1R+rq2R=[(2−d)(a−c+ε)+rd]28(2−d2) +r(4−d2−2d)(a−c+ε)−(4−d2)r4(2−d2).

Choosing *r* to maximize firm 1's total income yields

(19) $$\begin{array}{c} r=\frac{2\left(2-d^{2}\right)}{8-3d^{2}}\left(a-c+\varepsilon \right). \end{array}$$

### 4.1. Nondrastic Innovation Case

For the case of nondrastic innovation (i.e., *ε* < (*a* − *c*)(4 − *d* ^2^ − 2*d*)/2*d*), the maximum royalty rate that firm 1 can charge is such that firm 2's profit equals its no licensing profit, that is, *π*_2_^*R*^ = *π*_2_^NL^. From *π*_2_^*R*^ = *π*_2_^NL^, we have *r*_1_ = *ε*.

The optimal royalty rate is determined by taking the minimum of the rates r* = min⁡(*r*, *r*_1_). This is because when *r* < *r*_1_, it is in firm 1's best interest to charge *r* instead of *r*_1_, and when *r* ≥ *r*_1_, firm 1 is forced to charge *r*_1_ due to firm 2's acceptance constraint.

Comparing *r* and *r*_1_, we get the following: if

(20) $$\begin{array}{c} \varepsilon <\frac{2\left(2-d^{2}\right)}{4-d^{2}}\left(a-c\right), \end{array}$$

then the optimal royalty rate r* = *ε*; and if

(21) $$\begin{array}{c} \varepsilon \geq \frac{2\left(2-d^{2}\right)}{4-d^{2}}\left(a-c\right), \end{array}$$

then the optimal royalty rate r* = (2(2 − *d* ^2^)/(8 − 3*d* ^2^))(*a* − *c* + *ε*).

If (20) is satisfied, substituting r* = *ε* into (17), then we have

(22) q1R=(2−d)(a−c)+2ε2(2−d2),q2R=(4−d2−2d)(a−c)−2dε4(2−d2),

(23) π1R=[(2−d)(a−c)+2ε]28(2−d2),π2R=[(4−d2−2d)(a−c)−2dε]216(2−d2)2.

By (18), firm 1's total income is

(24) π1R+rq2R=[(2−d)(a−c)+2ε]28(2−d2) +(4−d2−2d)(a−c)ε−2dε24(2−d2).

Comparing (6), (23), and (24), we see that *π*_1_^*R*^ = *π*_1_^NL^ and *π*_2_^*R*^ = *π*_2_^NL^; then we have *π*_1_^*R*^ = *π*_1_^NL^ + *rq*_2_^*R*^ > *π*_1_^NL^. Thus, we can conclude licensing is better than not licensing for firm 1. Note that from (6) and (23), firm 2 is indifferent about licensing and not licensing from firm 1 in this case.

If (21) is satisfied, substituting r* = (2(2 − *d* ^2^)/(8 − 3*d* ^2^))(*a* − *c* + *ε*) into (17), then we have

(25) q1R=[(2−d)+2d(2−d2)/(8−3d2)](a−c+ε)2(2−d2),q2R=([(4−d2−2d)−2(4−d2)(2−d2)8−3d2](a−c+ε))  ×(4(2−d2))−1,

(26) π1R=([(2−d)+2d(2−d2)8−3d2]2(a−c+ε)2)8(2−d2),π2R=([(4−d2−2d)−2(4−d2)(2−d2)8−3d2]2×(a−c+ε)2)(16(2−d2)2)−1.

By (18), firm 1's total income is

(27) π1R+rq2R={[(2−d)+2d(2−d2)/(8−3d2)]28(2−d2)+2(4−d2−2d)(2−d2)/(8−3d2)4(2−d2)−(4−d2)[2(2−d2)/(8−3d2)]24(2−d2)} ×(a−c+ε)2.

Comparing (6) and (27), we can easily find that firm 1's total income given in (27) is greater than that given in (6). Hence, licensing is better than not licensing from the viewpoint of firm 1. Straightforward calculations show that firm 2's income given by (26) is no less than that given by (6). Hence, firm 2 is willing to accept the license from firm 1.

Hence, with a nondrastic innovation, firm 1 will always license its innovation to firm 2.

### 4.2. Drastic Innovation Case

For the case of drastic innovation (i.e., *ε* ≥ (*a* − *c*)(4 − *d* ^2^ − 2*d*)/2*d*), the maximum royalty rate that firm 1 can charge is such that firm 2's profit equals its no licensing profit, that is, *π*_2_^*R*^ = *π*_2_^NL^. We get that

(28) $$\begin{array}{c} r_{2}=\frac{4-d^{2}-2d}{4-d^{2}}\left(a-c+\varepsilon \right). \end{array}$$

The optimal royalty rate is determined by taking the minimum of the rates r* = min⁡⁡(*r*, *r*_2_).

Comparing *r* and *r*_2_, we get the following:

 
*r* < *r*_2_ when 0 < *d* < 0.9118 and *r* > *r*_2_ when *d* > 0.9118. Therefore, r* = *r* when 0 < *d* < 0.9118 and r* = *r*_2_ when *d* > 0.9118.

When 0 < *d* < 0.9118, substituting r* = *r* = (2(2 − *d* ^2^)/(8 − 3*d* ^2^))(*a* − *c* + *ε*) into (17), we find that firm 1 and firm 2's equilibrium quantities and profits are the same as (25) and (26). Hence, firm 1's total income is given by (27).

Comparing (27) and (8), we have

(29) $$\begin{array}{c} \pi_{1}^{R}+rq_{2}^{R}-\pi_{1}^{\text{NL}}=\left(V-\frac{1}{4}\right){\left(a-c+\varepsilon \right)}^{2}, \end{array}$$

where

(30) V=[(2−d)+(2d(2−d2))/(8−3d2)]28(2−d2) +2(4−d2−2d)(2−d2)/(8−3d2)4(2−d2) −(4−d2)[2(2−d2)/(8−3d2)]24(2−d2).

Standard computations yield that *π*_1_^*R*^ + *rq*_2_^*R*^ ≥ *π*_1_^NL^ when *d* ≤ 0.7476, and *π*_1_^*R*^ + *rq*_2_^*R*^ < *π*_1_^NL^ when 0.7476 < *d* < 0.9118. Hence, when *d* ≤ 0.7476, licensing by means of royalty will occur; when 0.7476 < *d* < 0.9118, firm 1 will not license its innovation to firm 2.

When *d* > 0.9118, substituting r* = *r*_2_ = ((4 − *d* ^2^ − 2*d*)/(4 − *d* ^2^))(*a* − *c* + *ε*) into (18), firm 1's total income is given by

(31) π1R+rq2R={[(2−d)+d(4−d2−2d)/(4−d2)]28(2−d2)+(4−d2−2d)(4−d2−2d)/(4−d2)4(2−d2)−(4−d2)[(4−d2−2d)/(4−d2)]24(2−d2)} ×(a−c+ε)2.

Comparing (31) and (8), we have

(32) $$\begin{array}{c} \pi_{1}^{R}+rq_{2}^{R}-\pi_{1}^{\text{NL}}=\left(W-\frac{1}{4}\right){\left(a-c+\varepsilon \right)}^{2}, \end{array}$$

where

(33) W=[(2−d)+d(4−d2−2d)/(4−d2)]28(2−d2) +(4−d2−2d)(4−d2−2d)/(4−d2)4(2−d2) −(4−d2)[(4−d2−2d)/(4−d2)]24(2−d2).

Straightforward calculations show that firm 1's total income given by (31) is less than that given by (8) (i.e., *π*_1_^*R*^ + *rq*_2_^*R*^ − *π*_1_^NL^ < 0) for *d* > 0.9118. Hence, firm 1 will not license its innovation to firm 2 in this case.

Hence, with a drastic innovation, we can state the following result. If 0 < *d* ≤ 0.7476 then firm 1 will license its innovation and if *d* > 0.7476 firm 1 will not license its innovation, and will become a monopoly.

We summarize the preceding results in Lemma 6.

**Lemma 6**

Under a royalty licensing method, firm 1 will always license its nondrastic innovation to firm 2; with a drastic innovation, if 0 < *d* ≤ 0.7476 firm 1 will license its innovation and if *d* > 0.7476 firm 1 will not license its innovation and will become a monopoly.

Wang [12] shows that, in a Cournot duopoly model, licensing will always occur under royalty licensing whether the innovation is nondrastic or drastic, while we find that the leader (innovator) will always license its nondrastic innovation to the follower under royalty licensing. However, with a drastic innovation, whether or not firm 1 licenses its innovation, it depends on the substitution coefficient *d* of the two goods. When the two goods are more differentiated (0 < *d* ≤ 0.7476) then it is more profitable for firm 1 to license its drastic innovation to firm 2. But, when the two goods are closer substitutes (*d* > 0.7476) then firm 1 will keep its drastic innovation and become a monopoly.

## 5. Two-Part Tariff Licensing

In this section, we consider licensing by means of a hybrid contract consisting of a fixed fee and a royalty. In this case, firm 1's marginal cost is *c*_1_ = *c* − *ε* and firm 2's marginal cost is *c*_2_ = *c* − *ε* + *r*. Substituting *c*_1_ = *c* − *ε* and *c*_2_ = *c* − *ε* + *r* into (3) and (4) yields the firms' equilibrium quantities and profits (the superscript RF denotes the case of “two-part tariff licensing”):

(34) q1RF=(2−d)(a−c+ε)+rd2(2−d2),q2RF=(4−d2−2d)(a−c+ε)−(4−d2)r4(2−d2),

(35) π1RF=[(2−d)(a−c+ε)+rd]28(2−d2),π2RF=[(4−d2−2d)(a−c+ε)−(4−d2)r]216(2−d2)2.

### 5.1. Nondrastic Innovation

With a nondrastic innovation (i.e., *ε* < (*a* − *c*)(4 − *d* ^2^ − 2*d*)/2*d*), the optimal fixed fee that firm 1 can charge is

(36) FRF=π2RF−π2NL=[(4−d2−2d)(a−c+ε)−(4−d2)r]216(2−d2)2 −[(4−d2−2d)(a−c)−2dε]216(2−d2)2.

Firm 1's total income is

(37) π1RF+rq2RF+FRF  =[(2−d)(a−c+ε)+rd]28(2−d2)   +r(4−d2−2d)(a−c+ε)−(4−d2)r4(2−d2)   +[(4−d2−2d)(a−c+ε)−(4−d2)r]216(2−d2)2   −[(4−d2−2d)(a−c)−2εd]216(2−d2)2.

Choosing *r* to maximize firm 1's total income yields

(38) $$\begin{array}{c} r_{1}=\frac{8d-8d^{2}-2d^{3}+3d^{4}}{16-20d^{2}+5d^{4}}\left(a-c+\varepsilon \right). \end{array}$$

The royalty rate *r*_1_ can maximize firm 1's profits regardless of firm 2's acceptance. In order to make firm 2 accept the license, we have to consider firm 2's acceptance constraint. We find the royalty rate where firm 2's acceptance constraint is binding (i.e., *π*_2_^RF^ = *π*_2_^NL^), denoted by *r*_2_. From *π*_2_^RF^ = *π*_2_^NL^, we can easily find that the maximum royalty rate firm 2 will agree to is *r*_2_ = *ε*.

Next, we will determine the optimal royalty rate *r*; the optimal royalty rate can be derived by taking the minimum of the rates *r* = min⁡⁡(*r*_1_, *r*_2_). This is because when *r*_1_ < *r*_2_, firm 1 can gain the most profits by charging *r*_1_, and when *r*_1_ ≥ *r*_2_, firm 1 is forced to charge *r*_2_ due to firm 2's acceptance constraint.

Comparing with *r*_1_ and *r*_2_, we have the following: if

(39) $$\begin{array}{c} \varepsilon \geq \frac{8d-8d^{2}-2d^{3}+3d^{4}}{2\left(8-4d-6d^{2}+d^{3}+d^{4}\right)}\left(a-c\right), \end{array}$$

then the optimal royalty rate *r* = *r*_1_; and if

(40) $$\begin{array}{c} \varepsilon <\frac{8d-8d^{2}-2d^{3}+3d^{4}}{2\left(8-4d-6d^{2}+d^{3}+d^{4}\right)}\left(a-c\right), \end{array}$$

then the optimal royalty rate *r* = *r*_2_ = *ε*.

If (39) holds, substituting *r* = ((8*d* − 8*d* ^2^ − 2*d* ^3^ + 3*d* ^4^)/(16 − 20*d* ^2^ + 5*d* ^4^))(*a* − *c* + *ε*) into (37), we have

(41) π1RF+rq2RF+FRF={([(2−d)+d(8d−8d2−2d3+3d4)16−20d2+5d4]2×(8(2−d2))−1)+([(4−d2−2d)(8d−8d2−2d3+3d4)16−20d2+5d4]−(4−d2)[8d−8d2−2d3+3d416−20d2+5d4]2)×(4(2−d2))−1+([(4−d2−2d)−(4−d2)(8d−8d2−2d3+3d4)16−20d2+5d4]2×(16(2−d2)2)−1)}(a−c+ε)2 −[(4−d2−2d)(a−c)−2εd]216(2−d2)2.

Comparing *π*_1_^NL^ in (6) and (41), we can easily find that firm 1's total income given in (41) is greater than that given in (6). Hence, licensing is better than not licensing from the viewpoint of firm 1. Straightforward calculations show that firm 2's income given by (35), for *r* = *r*_1_, is no less than that given by (6). Hence, firm 2 is willing to accept the license from firm 1.

If (40) holds, substituting *r* = *ε* into (37), we have

(42) π1RF+rq2RF+FRF=[(2−d)(a−c+ε)+dε]28(2−d2) +ε[(4−2d−d2)(a−c)−2dε]4(2−d2).

Comparing *π*_1_^NL^ in (6) and (42), we can easily find that firm 1's total income given in (42) is greater than that given in (6). Hence, licensing is better than not licensing from the viewpoint of firm 1. Straightforward calculations show that firm 2's income given by (35), for *r* = *ε*, is equal to that given by (6). Hence, firm 2 is willing to accept the license from firm 1.

Hence, with a nondrastic innovation, firm 1 will always license its innovation to firm 2.

### 5.2. Drastic Innovation

With a drastic innovation (i.e., *ε* ≥ (*a* − *c*)(4 − *d* ^2^ − 2*d*)/2*d*), the optimal fixed fee that firm 1 can charge is

(43) FRF=π2RF−π2NL=[(4−d2−2d)(a−c+ε)−(4−d2)r]216(2−d2)2.

Firm 1's total income is

(44) π1RF+rq2RF+FRF  =[(2−d)(a−c+ε)+rd]28(2−d2)   +r(4−d2−2d)(a−c+ε)−(4−d2)r4(2−d2)   +[(4−d2−2d)(a−c+ε)−(4−d2)r]216(2−d2)2.

Choosing *r* to maximize firm 1's total income yields

(45) $$\begin{array}{c} r_{1}=\frac{8d-8d^{2}-2d^{3}+3d^{4}}{16-20d^{2}+5d^{4}}\left(a-c+\varepsilon \right). \end{array}$$

From *π*_2_^RF^ = *π*_2_^NL^, we find that the maximum royalty rate firm 2 can afford is

(46) $$\begin{array}{c} r_{2}=\frac{4-d^{2}-2d}{4-d^{2}}\left(a-c+\varepsilon \right). \end{array}$$

Let *A* = ((4 − *d* ^2^ − 2*d*)/(4 − *d* ^2^))−((8*d* − 8*d* ^2^ − 2*d* ^3^ + 3*d* ^4^)/(16 − 20*d* ^2^ + 5*d* ^4^)), then we can easily find *A* > 0, when *d* < 0.9118; *A* ≤ 0, when *d* ≥ 0.9118.

Hence, the optimal royalty rate is *r* = *r*_1_ when *d* < 0.9118; the optimal royalty rate is *r* = *r*_2_ when *d* ≥ 0.9118.

When *d* < 0.9118, substituting *r* = ((8*d* − 8*d* ^2^ − 2*d* ^3^ + 3*d* ^4^)/(16 − 20*d* ^2^ + 5*d* ^4^))(*a* − *c* + *ε*) into (44), we have

(47) π1RF+rq2RF+FRF={([(2−d)+d(8d−8d2−2d3+3d4)16−20d2+5d4]2×(8(2−d2))−1)+(([(4−d2−2d)(8d−8d2−2d3+3d4)16−20d2+5d4]−(4−d2)[8d−8d2−2d3+3d416−20d2+5d4]2)×(4(2−d2))−1)+([(4−d2−2d)−(4−d2)(8d−8d2−2d3+3d4)16−20d2+5d4]2×(16(2−d2)2)−1)} ×(a−c+ε)2.

Comparing *π*_1_^NL^ in (8) and (47), we have

(48) $$\begin{array}{c} \pi_{1}^{\text{RF}}+rq_{2}^{\text{RF}}+F^{\text{RF}}-\pi_{1}^{\text{NL}}=\left(X-\frac{1}{4}\right){\left(a-c+\varepsilon \right)}^{2}, \end{array}$$

where

(49) X=([(2−d)+d(8d−8d2−2d3+3d4)16−20d2+5d4]2×(8(2−d2))−1)+([(4−d2−2d)(8d−8d2−2d3+3d4)16−20d2+5d4]−(4−d2)[8d−8d2−2d3+3d416−20d2+5d4]2×(4(2−d2))−1)+([(4−d2−2d)−(4−d2)(8d−8d2−2d3+3d4)16−20d2+5d4]2×(16(2−d2)2)−1).

Straightforward calculations show that firm 1's total income given by (47) is greater than that given by (8) for 0 < *d* < 0.8061. Hence, licensing is better than not licensing for firm 1. Further, we can also verify that firm 2 is better off in case of licensing than not licensing from firm 1. However, firm 1's total income given by (47) is less than that given by (8) for 0.8061 < *d* < 0.9118. Hence, not licensing is better than licensing for firm 1.

When *d* ≥ 0.9118, substituting *r* = ((4 − *d* ^2^ − 2*d*)/(4 − *d* ^2^))(*a* − *c* + *ε*) into (44), we have

(50) π1RF+rq2RF+FRF={([(2−d)+d(4−d2−2d)4−d2]2(8(2−d2))−1)+([(4−d2−2d)(4−d2−2d)4−d2]−(4−d2)[4−d2−2d4−d2]2(4(2−d2))−1)+([(4−d2−2d)−(4−d2)(4−d2−2d)4−d2]2×(16(2−d2)2)−1)} ×(a−c+ε)2.

Comparing *π*_1_^NL^ in (8) and (50), we have

(51) $$\begin{array}{c} \pi_{1}^{\text{RF}}+rq_{2}^{\text{RF}}+F^{\text{RF}}-\pi_{1}^{\text{NL}}=\left(Y-\frac{1}{4}\right){\left(a-c+\varepsilon \right)}^{2}, \end{array}$$

where

(52) Y=([(2−d)+d(4−d2−2d)4−d2]2(8(2−d2))−1) +([(4−d2−2d)(4−d2−2d)4−d2]−(4−d2)[4−d2−2d4−d2]2×(4(2−d2))−1) +([(4−d2−2d)−(4−d2)(4−d2−2d)4−d2]2×(16(2−d2)2)−1).

Straightforward calculations show that firm 1's total income given by (50) is less than that given by (8) for *d* ≥ 0.9118. Hence, firm 1 will not license its innovation to firm 2.

Hence, with a drastic innovation, licensing is better for both parties if 0 < *d* < 0.8061. However, firm 1 is worse off if *d* ≥ 0.8061; in this case firm 1 will keep its innovation for its own use and become a monopoly.

To summarize the above results, we have the following conclusion.

**Lemma 7**

Under a two-part tariff licensing method, firm 1 will always license its nondrastic innovation to firm 2. With a drastic innovation, firm 1 will license its innovation to firm 2 if 0 < *d* < 0.8061 and will not license it if *d* ≥ 0.8061.

## 6. Comparison of the Three Licensing Methods

### 6.1. Comparison: Fixed-Fee versus Royalty Licensing

In this subsection, we will compare fixed-fee licensing and royalty licensing to decide which licensing method is better.

Consider first a nondrastic innovation (i.e., *ε* < (*a* − *c*)(4 − *d* ^2^ − 2*d*)/2*d*). If (20) holds, comparing (12) and (24), we have the following:

(53) (π1F+F)−(π1R+rq2R)=−d(8−8d−2d2+3d3)16(2−d2)2(a−c)ε +16−16d−4d2+4d3−d416(2−d2)2ε2.

Let (*π*_1_^*F*^ + *F*)−(*π*_1_^*R*^ + *rq*_2_^*R*^) < 0; then we have

(54) $$\begin{array}{c} \varepsilon <\frac{8d+8d^{2}-2d^{3}+3d^{4}}{16-16d-4d^{2}+4d^{3}-d^{4}}\left(a-c\right). \end{array}$$

Let *B* = ((8*d* + 8*d* ^2^ − 2*d* ^3^ + 3*d* ^4^)/(16 − 16*d* − 4*d* ^2^ + 4*d* ^3^ − *d* ^4^))−((2(2 − *d* ^2^))/(4 − *d* ^2^)), then we can easily find *B* < 0, when *d* < 0.5290; *B* ≥ 0, when 0.5290 ≤ *d* < 0.9380.

Lemma 5 indicates that firm 1 will license its nondrastic innovation to firm 2 if and only if 0 < *d* ≤ 0.7801. Hence, if (54) holds for *d* < 0.5290, licensing by means of a royalty is superior to licensing by means of a fee for firm 1 if *d* < 0.5290. For the case of *d* ≥ 0.5290, we also find licensing by means of a royalty is superior to licensing by means of a fee. Hence, if (20) holds, licensing by means of a royalty is always superior to licensing by means of a fee.

If (21) holds, comparing (12) and (27), we have the following:

(55) (π1F+F)−(π1R+rq2R)  =[(4−d2−4d)(4−d2)16(2−d2)2+(2−d)28(2−d2)−V]ε2   +[(2−d)28(2−d2)−V](a−c)2   +2[(2−d)28(2−d2)+(4−d2−2d)(4−d2)16(2−d2)2−V]   +(a−c)ε.

Straightforward calculations show (*π*_1_^*F*^ + *F*)>(*π*_1_^*R*^ + *rq*_2_^*R*^), if 0.5160 < *d* ≤ 0.7801. Hence, licensing by means of a fee is superior to licensing by means of a royalty. From Lemma 5, we can easily find that fixed-fee licensing will not occur for *d* > 0.7801, so licensing by means of a royalty is superior to licensing by means of a fee for firm 1.

Consider next a drastic innovation (i.e., *ε* ≥ (*a* − *c*)(4 − *d* ^2^ − 2*d*)/2*d*).

With a drastic innovation, licensing does not occur under fee licensing, while royalty licensing will occur if 0 < *d* ≤ 0.7476. For the case of *d* > 0.7476, fee and royalty licensing will not occur because the necessary conditions are absent.

To summarize the above results, we obtain the following proposition.

**Proposition 8**

*(1)* With a nondrastic innovation, licensing by means of a royalty is always superior to licensing by means of a fee for firm 1. *(2)* With a drastic innovation, licensing by means of a royalty is superior to licensing by means of a fee for firm 1 if 0 < *d* ≤ 0.7476; if *d* > 0.7476 then neither fee nor royalty licensing will occur and firm 1 will become a monopoly.

Case (1) in Proposition 8 demonstrates that firm 1 will always license its nondrastic innovation to firm 2 for all *d*: 0 < *d* < 1. This conclusion is different from Wang [12] which purports that licensing by means of a fee dominates licensing by means of a royalty for firm 1 when both the substitution coefficient and the magnitude of the innovation are small. This conclusion is also in contrast with the result in Kamien and Truman [5], in which the licensor is an outside innovator and it licenses a cost-reducing innovation to one or both downstream firms by means of either a fee or a royalty. Their result shows that licensing by means of a fee dominates licensing by means of a royalty.

Case (2) in Proposition 8 demonstrates that in the case of drastic innovation royalty licensing is better than fee licensing for firm 1 when the substitution coefficient *d* is small. This result is completely different from Wang [12]. Wang [12] also purports that, when both fixed-fee licensing and royalty licensing are considered, firm 1 will always license its drastic innovation. In our work, however, we find that both fixed-fee licensing and royalty licensing will not occur for large values of *d*. The cause for these contrasting results lies in the fact that the competition pattern in the present model is different from that of Wang. Wang [12] considers the licensing in a duopoly market where the decisions are made simultaneously (Cournot model), while, in the present paper, we consider the licensing in a duopoly market where the decisions are made sequentially (Stackelberg model).

### 6.2. Comparison: Fixed-Fee Licensing versus Two-Part Tariff Licensing

In this subsection, we will compare fixed-fee licensing and two-part tariff licensing to decide which licensing method is better.

Consider first a nondrastic innovation (i.e., *ε* < (*a* − *c*)(4 − *d* ^2^ − 2*d*)/2*d*). If (39) holds, comparing (12) and (41), we have the following:

(56) (π1RF+rq2RF+FRF)−(π1F+F) =[X−(2−d)28(2−d2)−(4−d2−2d)216(2−d2)2](a−c+ε)2.

From Lemma 5, we know firm 1 will not license its nondrastic innovation to firm 2 if *d* > 0.7801; under this situation, two-part tariff licensing always dominates fixed-fee licensing. It is easy to check that (*π*_1_^RF^ + *rq*_2_^RF^ + *F* ^RF^)−(*π*_1_^*F*^ + *F*) > 0 is always satisfied when *d* ≤ 0.780054.

Consider next a drastic innovation (i.e., *ε* ≥ (*a* − *c*)(4 − *d* ^2^ − 2*d*)/2*d*). In this situation, fixed-fee licensing will not occur; hence, two-part tariff licensing is better than fixed-fee licensing for firm 1.

We summarize the above results in the following proposition.

**Proposition 9**

Two-part tariff licensing is always superior to fixed-fee licensing.

### 6.3. Comparison: Royalty Licensing versus Two-Part Tariff Licensing

In this subsection, we will compare royalty licensing and two-part tariff licensing to decide which licensing method is better.

Consider first a nondrastic innovation (i.e., *ε* < (*a* − *c*)(4 − *d* ^2^ − 2*d*)/2*d*). Under this condition, there are two optimal royalty rates depending on whether (20) or (21) is satisfied. Hence, there are three scenarios to analyze in order to compare the two alternative licensing methods.

Let *f* = ((8*d* − 8*d* ^2^ − 2*d* ^3^ + 3*d* ^4^)/(2(8 − 4*d* − 6*d* ^2^ + *d* ^3^ + *d* ^4^))) − ((2(2 − *d* ^2^))/(4 − *d* ^2^)). Straightforward calculations show that *f* ≤ 0, if 0 ≤ *d* ≤ 0.911773; *f* ≥ 0, if 0.911773 < *d* ≤ 1.

Under the condition of 0 ≤ *d* ≤ 0.911773, if (20) and (39) hold simultaneously, then we need to compare (24) and (41). If the optimal royalty rate *r* = ((8*d* − 8*d* ^2^ − 2*d* ^3^ + 3*d* ^4^)/(16 − 20*d* ^2^ + 5*d* ^4^))(*a* − *c* + *ε*), firm 1's total income under two-part tariff licensing is given by (41) which is obviously greater than *π*_1_^*R*^ + *rq*_2_^*R*^ in (24). Hence, two-part tariff licensing is better than royalty licensing. If (21) helds, then (39) must be hold. We need to compare (41) and (27):

(57) (π1RF+rq2RF+FRF)−(π1R+rq2R)  =[X−V−(4−d2−2d)216(2−d2)2](a−c)2   +2[X−V+2d(4−d2−2d)16(2−d2)2](a−c)ε   +[X−V−4d216(2−d2)2]ε2.

Straightforward numerical calculations show that, if *d* ≤ 0.9118, then two-part tariff licensing is better than royalty licensing; if *d* > 0.9118, then the condition of nondrastic innovation under royalty licensing does not hold; hence, two-part tariff licensing is better than royalty licensing.

Consider next a drastic innovation (i.e., *ε* ≥ (*a* − *c*)(4 − *d* ^2^ − 2*d*)/2*d*). Under this condition, firm 1 will license its innovation by choosing the optimal licensing method from royalty and two-part tariff only if *d* < 0.7476. In order to derive which licensing method is better, we need to solve the following problem:

(58) $$\begin{array}{c} \left(\pi_{1}^{\text{RF}}+rq_{2}^{\text{RF}}+F^{\text{RF}}\right)-\left(\pi_{1}^{R}+rq_{2}^{R}\right)=\left(X-V\right){\left(a-c+\varepsilon \right)}^{2}. \end{array}$$

If *d* < 0.7476 is satisfied, firm 1's total income under two-part tariff licensing is greater than that under royalty licensing by straightforward numerical calculations, that is, (*π*_1_^RF^ + *rq*_2_^RF^ + *F* ^RF^)−(*π*_1_^*R*^ + *rq*_2_^*R*^) ≥ 0; hence, two-part tariff licensing is better than royalty licensing for firm 1. On the contrary, royalty licensing is better than two-part tariff licensing for firm 1 if 0.7476 < *d* < 1.

To summarize the above results, we obtain the following proposition.

**Proposition 10**

(1) With a nondrastic innovation, two-part tariff licensing is always superior to royalty licensing for firm 1. (2) With a drastic innovation, licensing by means of a two-part tariff is superior to licensing by means of a royalty for firm 1 if 0 < *d* ≤ 0.7476; if *d* > 0.7476 then we can get an opposite conclusion.

## 7. Conclusions

This paper extends Wang's [12] duopoly differentiated Cournot model to a duopoly differentiated Stackelberg model, in which an inside innovator has a patent over a cost-reducing innovation. The focus of the present paper is on the choices of the patent-holding firm's optimal licensing strategy. To this end, we study and compare the three popular licensing methods.

The main findings of this paper are as follows. (1) With a nondrastic innovation, royalty licensing is always superior to fixed-fee licensing for the innovator; with a drastic innovation, royalty licensing is better than fixed-fee licensing for small values of *d*; however when *d* becomes closer to 1, neither fee nor royalty licensing will occur. (2) The innovator is always better off in case of two-part tariff licensing than fixed-fee licensing no matter what the magnitude of the innovation is. (3) It is more advantageous for the innovator to license its nondrastic innovation by means of two-part tariff licensing than royalty licensing; with a drastic innovation, we find that the degree of the differentiation of the goods plays an important role in the results. These conclusions are different from, Wang [12].

Our study leaves several unanswered questions for future research. Firstly, we assume that licensing decreases the licensee's marginal cost to the same level as that of the licensor. Future research can relax this assumption to take into account the asymmetry of the production cost between the licensor and the licensee in a differentiated Stackelberg model. Secondly, we assume that the licensor makes a take-it-or-leave-it offer to the licensee. However, in practice, it is often observed that the licensor and the licensee negotiate for licensing the patent. Thus, future research can study technology licensing from the cooperative game theoretic viewpoint to deeply explore the licensing behavior with side payments between the licensor and the licensee. Thirdly, there is only one licensee in this paper. Therefore another interesting extension to our research would be to investigate the effect of competition among *n* (*n* ≥ 2) licensees on the optimal licensing strategy.
